# Concept and modelling of memsensors as two terminal devices with enhanced capabilities in neuromorphic engineering

**DOI:** 10.1038/s41598-019-39008-5

**Published:** 2019-03-13

**Authors:** Alexander Vahl, Jürgen Carstensen, Sören Kaps, Oleg Lupan, Thomas Strunskus, Rainer Adelung, Franz Faupel

**Affiliations:** 1 Institute for Materials Science – Chair for Multicomponent Materials, Faculty of Engineering, Christian-Albrechts-University of Kiel, Kaiserstraße 2, D-24143 Kiel, Germany; 2 Institute for Materials Science – Functional Nanomaterials, Faculty of Engineering, Christian-Albrechts-University of Kiel, Kaiserstraße 2, D-24143 Kiel, Germany; 3 Department of Microelectronics and Biomedical Engineering, Center for Nanotechnology and Nanosensors, Technical University of Moldova, 168 Stefan cel Mare Av., MD-2004 Chisinau, Republic of Moldova

## Abstract

We report on memsensors, a class of two terminal devices that combines features of memristive and sensor devices. Apart from a pinched hysteresis (memristive property) and stimulus dependent electrical resistance (sensing property) further properties like dynamic adaptation to an external stimulus emerge. We propose a three component equivalent circuit to model the memsensor electrical behaviour. In this model we find stimulus dependent hysteresis, a delayed response to the sensory signal and adaptation. Stimulus dependent IV hysteresis as a fingerprint of a memsensor device is experimentally shown for memristive ZnO microrods. Adaptation in memsensor devices as found in our simulations resembles striking similarities to the biology. Especially the stimulus dependency of the IV hysteresis and the adaptation to external stimuli are superior features for application of memsensors in neuromorphic engineering. Based on the simulations and experimental findings we propose design rules for memsensors that will facilitate further research on memsensitive systems.

## Introduction

Since the postulation of the experimental realization of a memristor device in 2008^1^, there has been an increased effort in understanding and modelling memristive (resistive) switching for a broad variety of device classes^2–4^. Up to now (2018) an increasing group of concepts for memristive devices with sensing behaviour has been reported, typically based on metal oxide or semiconductor nanostructures with sensing of temperature^5^, (UV)-light^6–9^, gases^10,11^, magnetic field^7^, mechanical response^12^, biomolecules^13–15^ or pH^16,17^. However, a systematic treatment of the junction between memristive and sensitive devices is still missing.

In this work we report on the concept of memsensors, a class of two terminal electronic devices that combine the properties of memristive switching and sensing properties. By thoughtfully designing electronic devices with both, a sensing part (stimulus dependent resistance) and a memristive part (pinched hysteresis and memory) it is possible to make use of their enhanced capabilities. In classical electronics respective controlling theory, sensors that show a hysteresis are typically regarded as a poor sensing device. However, features like adaptation and forgetting are essential for efficient learning in biological systems such as neuronal networks^18,19^. In memsensors, the combination of sensing and memory resembles the dynamic response of biological systems to environmental stimuli. One example is adaptation: the response to a constant excitation decreases asymptotically over time, the system accommodates to the constant stimulus. After reducing the stimulus for a long period, the system recovers its initial sensitivity. Adaptation is key for efficient use of neuronal capabilities^18^. First indications for adaptation in memsensor devices, although not specifically mentioned, can be found in the work of Chiolerio *et al*. and Lupan *et al*.^6,11^ Although over the past years there has been an increasing number of detailed reports on memristive switching devices combined with sensing capabilities, to the best of our knowledge a systematic description of the concept of memsensors as well as the link to adaptation is still missing.

In the current work, we deduct design guidelines to facilitate further research on memsensors based on the simulations and experimental findings.

## Overview on Memristive Devices with Sensing Capability

**Figure 1:**
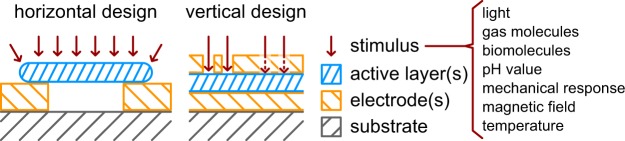
Possible device schematics (horizontal and vertical design) for memsensors as well as typically applied stimulus signals (e.g. light, etc.). For the horizontal design, the active layer (e.g. nanowire) is open to any stimulus. In the vertical design, the top electrode must not prevent the stimulus from reaching the active layer. This can be achieved by structuring (e.g. for gas molecules) or the choice of a transparent electrode material (e.g. ITO for light).

In 2008 Strukov *et al*. presented resistive switching in a TiO_x_ based device as the first realization of a memristor as predicted by Chua in 1971^1^. In the following years there were broad investigations on the understanding of memristive switching. The combination of memristive switching and sensing properties in the same device started with consideration of temperature sensitivity of memristive devices, e.g. by Wang *et al*. 2010 in their spintronic memristor^5,20^. The first experimental realizations of memristive devices mended with sensitive properties was achieved in 2012 by two groups: Ungureanu *et al*. reported on a light sensitive Si/Al_2_O_3_ stack and Carrara *et al*. presented a device based on single Si nanowires that showed capabilities of being used as a bio-sensor^13,21^. The latter concept was extended in a follow up work by Tzouvadaki and Carrara *et al*. to a memristive label free aptasensor with high sensitivity^15^. The term memsensor was, to the best of our knowledge, mentioned first in 2013 by Fan *et al*. in their work on mechanically stimulated CuO nanowires^12^. In 2013, Hadis *et al*. discussed various preparation routes and possible micro-structuring for memristive sensors based on the example of TiO_2_ for biosensing^14^. Already these early reports show a common feature: A prerequisite for all memsensor devices is their accessibility to the external stimulus. This condition is readily fulfilled for structures like nanowires, which are typically not covered by any contact or capping layer. In case of vertically stacked devices, where the contacts are applied at the bottom and the top of the active layer, the top layer would block signals like light, biomolecules or gases. For these devices, typically micro- and nanopatterning techniques like lithography are used to structure the top electrode in order to achieve sensing properties. The common design strategies as well as typically applied stimulus signals are depicted in Fig. 1.

Cross bar arrays of vertical memsensitive devices were realized by Nyenke *et al*. in 2016, using a layer of sub-stoichiometric copper oxide and patterned holes in the top electrode for oxygen sensing^10^. The combination of two sensing stimuli in a single device was achieved by Li *et al*. in 2014 in their light and magnetic field sensitive memristive device^7^. In 2016, Zhu *et al*. reported on a crossbar-array of stacked sensitive and memristive elements, which resembles features of an electronic skin^22^. In a closely related approach, Chen *et al*. used an array of In_2_O_3_ nanowires and Ni/Al_2_O_3_/Au memristive switches to design a visual memory system in 2018^9^.

## Results and Discussion

### The concept of a memsensor

**Figure 2:**
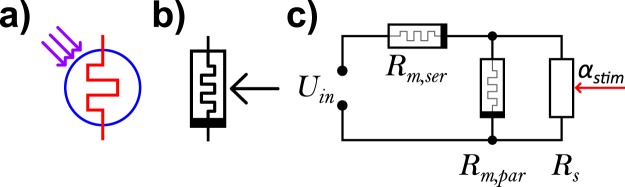
(**a**) Circuit symbol for a UV memsensor as proposed by Chiolerio *et al*.^6^; (**b**) generalized memsensor circuit symbol in agreement with the proposed (**c**) equivalent circuit of a memsensor device featuring memristive elements parallel (with *R*_*m,par*_) and in series (with *R*_*m,ser*_) to the sensing element (with *R*_*s*_). The resistance *R*_*s*_ is sensitive to an external stimulus *α*_*stim*_, e.g. gas concentration or UV-irradiation.

A circuit element for a light controlled memsensor acting as a current source was, to the best of our knowledge, first proposed by Chiolerio *et al*. in 2015 (Fig. 2a)^6^. Based on this, we propose a generalized circuit element for any memsensor that is sensitive to any kind of external stimulus (Fig. 2b). Although not addressed specifically in their work, early indications of adaptation to external stimulus appeared in the electrical characterization of the devices of Chiolerio *et al*. and Lupan *et al*.^6,11^.

**Figure 3:**
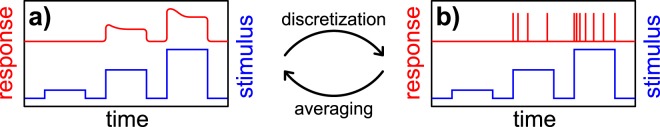
Adaptation to an external stimulus: (**a**) Amplitude adaptation: Amplitude of the response is proportional to the stimulus. (**b**) Spike frequency adaptation as proposed by E.D. Adrian in 1926: The frequency of spikes is proportional to the stimulus and fades over time. Spike frequency adaptation has been experimentally realized in a memristive spiking neuron circuit^23–25,37^. The system does not respond to subthreshold stimulus.

Common to all memsensitive devices discussed in the previous section is the influence of the external stimulus on the electronic structure. Accordingly, the memristive device is expanded by a sensing capability for this external stimulus. In addition to the core feature pinched hysteresis and stimulus dependent response a memsensor may show further features. In this paper we report on adaptation as an emergent feature of a memsensor. Adaptation in case of a memsensor describes the asymptotically decrease of the memsensor response to a constant stimulus after an initial high response (Fig. 3a).

The adaptation behaviour of a memsensor device shows parallels to adaptation in biology^18,23–25^. In the biological context adaptation is typically discussed with regard to firing frequency in pulsed operation (Fig. 3b). In case of spike frequency adaptation, the number of spikes per unit time (spike frequency) is proportional to the strength of the stimulus (a certain threshold stimulus may be overcome in order to excite spiking). For a constant applied stimulus the frequency of spikes decreases with time – the system adapts to the stimulus. Adaptation is believed to be an essential process of a signalling system to be better suited to environmental changes. It can be found in nearly all biological systems. Adaptation enables the system to blank out background signals and offers the possibility to be more sensitive to abrupt changes. Macroscopic effects of this adaptation process are for example the habituation to certain smells that are only perceived by the individual for a short time after entering a room. In fact, such complex habituation processes are not realized by a single neuron but involve the mutual interplay between various neuron ensembles, so called neural networks^26,27^.

In this paper we will show memsensors with adaptation in constant voltage mode with pulsed stimuli. Accordingly, the adaptation is in the amplitude of the memsensor response. This roughly relates to the integration over spike frequency modulated signal. However, this leads to an easier implementation to neuromorphic networks due to constant applied voltage. Accordingly, two terminal memsensor devices have high potential for neuromorphic engineering, e.g., as a feed forward network with input from external stimulus. For this purpose, sensory signals that can be applied locally are favourable, eliminating signals like temperature for the design of memsensors.

### Modelling of a memsensor device

In order to model a memsensor device that is capable of adaptation to the sensory signal, we propose a simple two terminal equivalent circuit (Fig. 2c) based on memristive elements in parallel (with *R*_*m,par*_) and in series (with *R*_*m,ser*_) to the sensing element (with *R*_*s*_).

The resistance of the sensory element *R*_*s*_ depends on the signal (external stimulus) that is supplied to the sensor. For an ideal linear sensor the resistivity *R*_*s*_ varies linearly between a high resistive state (HRS) and a low resistive state (LRS) Equation (1).

1 $${R}_{s}({\alpha }_{stim})={R}_{s,LRS}+(\frac{{\alpha }_{stim}}{{\alpha }_{stim,\max }})\cdot ({R}_{s,HRS}-{R}_{s,LRS})$$

For the memristive elements we approximate a simple linear dependence of the resistance on an internal state variable (*ω*) and the respective HRS and LRS of the memristive element (Equation (2)). Conventionally the memristive element is in its high resistance state in case the internal state variable equals zero.

2 $${R}_{m}(\omega )={R}_{m,LRS}+(1-\omega )\cdot ({R}_{m,HRS}-{R}_{m,LRS})$$

Based on the equivalent circuit, for any applied voltage *U*_*in*_, the individual potential drops *U*_*m*_ over the serial (Equation (3)) and the parallel (Equation (4)) memristive element are given.

3 $${U}_{m,ser}={U}_{in}\cdot \frac{{R}_{m,ser}}{{R}_{m,ser}+\frac{{R}_{m,par}\cdot {R}_{s}}{{R}_{m,par}+{R}_{s}}}$$

4 $${U}_{m,par}={U}_{in}\cdot \frac{\frac{{R}_{m,par}\cdot {R}_{s}}{{R}_{m,par}+{R}_{s}}}{{R}_{m,ser}+\frac{{R}_{m,par}\cdot {R}_{s}}{{R}_{m,par}+{R}_{s}}}$$

In our simulation the internal state variable for each memristive element is updated in each time step and the respective resistances and voltages are calculated accordingly. We chose a model for the memristive device (Equation (5)) in which the internal state variable *ω* is changed depending on the polarity of applied voltage, a threshold voltage *U*_*s*_ and a constant internal back driving voltage *U*_*b*_, representing all internal forces driving the system back into its equilibrium state (which is in our model the HRS). The back driving voltage *U*_*b*_ relates to the sum of all processes that relax the system into its equilibrium, comparable to the back driving (restoring) force in common Boltzmann-equation-like approaches^28^. These potentials translate to an electrical field across the device that results in forces on the electrically charged mobile species (e.g. cations, oxygen vacancies) in the memristive elements. The memristive device switches to its LRS if it is subjected to a voltage that is higher than the threshold voltage and the back driving voltage. This assumption of a voltage driven model with a threshold voltage and backdriving voltage is in close relation to memristive switching that relies on metal cation movement. In such electrochemical metallization cells^3^ the ionization processes, which are necessary to oxidize the metal atoms from the active electrode, resemble a threshold. The applied voltage drives the transport of metal cations through the matrix. The back driving force and the time constant *τ* define the retention time of the state of the memristive device.

5 $${\omega }_{i+1}=\{\begin{array}{ccc}{\omega }_{i}+\frac{|{U}_{m}|-{U}_{s}}{\tau }\cdot \frac{{U}_{m}}{|{U}_{m}|}-\frac{{U}_{b}}{\tau } & {\rm{for}} & |{U}_{m}|\ge {U}_{s}\\ {\omega }_{i}-\frac{{U}_{b}}{\tau } & {\rm{for}} & |{U}_{m}| < {U}_{s}\,{\rm{and}}\,0\le {\omega }_{i}-\frac{{U}_{b}}{\tau }\,\\ 1 & {\rm{for}} & 1 < {\omega }_{i}+\frac{1}{\tau }\cdot (|{U}_{m}|-{U}_{s})\cdot \frac{{U}_{m}}{|{U}_{m}|}-\frac{{U}_{b}}{\tau }\\ 0 & {\rm{for}} & 0 > {\omega }_{i}+\frac{1}{\tau }\cdot (|{U}_{m}|-{U}_{s})\cdot \frac{{U}_{m}}{|{U}_{m}|}-\frac{{U}_{b}}{\tau }\,{\rm{or}}\,0 > {\omega }_{i}-\frac{{U}_{b}}{\tau }\end{array}$$

The built-in back driving potential and the threshold voltage both prevent the system from switching to LRS at low voltages. Equation (5) may be substantially simplified in case there is no threshold voltage (Equation (6)).

6 $${\omega }_{i+1}=\{\begin{array}{ccc}{\omega }_{i}+\frac{{U}_{m}-{U}_{b}}{\tau } & {\rm{for}} & 0\le {\omega }_{i}+\frac{{U}_{m}-{U}_{b}}{\tau }\le 1\\ 1 & {\rm{for}} & 1 < {\omega }_{i}+\frac{{U}_{m}-{U}_{b}}{\tau }\\ 0 & {\rm{for}} & 0 > {\omega }_{i}+\frac{{U}_{m}-{U}_{b}}{\tau }\end{array}\,\,$$

**Figure 4:**
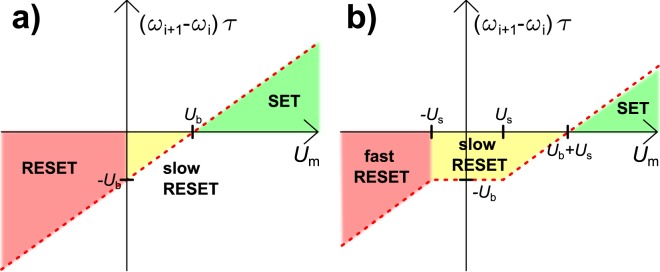
Schematic overview over the switching regimes realized by the mathematical representation of the memristive device in Equation (6) (**a**) and Equation (5) (**b**). The SET regime (green) corresponds to a switching of the memristive device to LRS at voltages above the threshold voltage. The slow RESET (yellow) describes the finite retention of the LRS. The fast RESET (red) below the negative threshold voltage corresponds to the switching to HRS.

The corresponding switching behaviour for a single memristive element is visualized in Fig. 4, once for the case without threshold voltage (Fig. 4a) and once for the more complex description (Fig. 4b). In both cases three distinct switching regimes are to be distinguished. SET for switching of the memristive device to low resistive state. Slow RESET, which is limiting the device retention time, as observed for a multitude of memristive systems. (Fast) RESET occurs at negative voltage over the threshold voltage for switching to HRS.

In order to overcome the necessity of defining additional conditions for accounting for the boundary cases of $\omega =1$ and $\omega =0$, the model may be extended by incorporation of a window function. Appropriate window functions for memristive models are discussed in the work of Prodromakis *et al*. and Zha *et al*.^29–31^. However, in this model we do not make use of any window function in order to keep a simple representation of all parameters by physical quantities.

### Hysteresis and adaptation in memsensor devices

**Figure 5:**
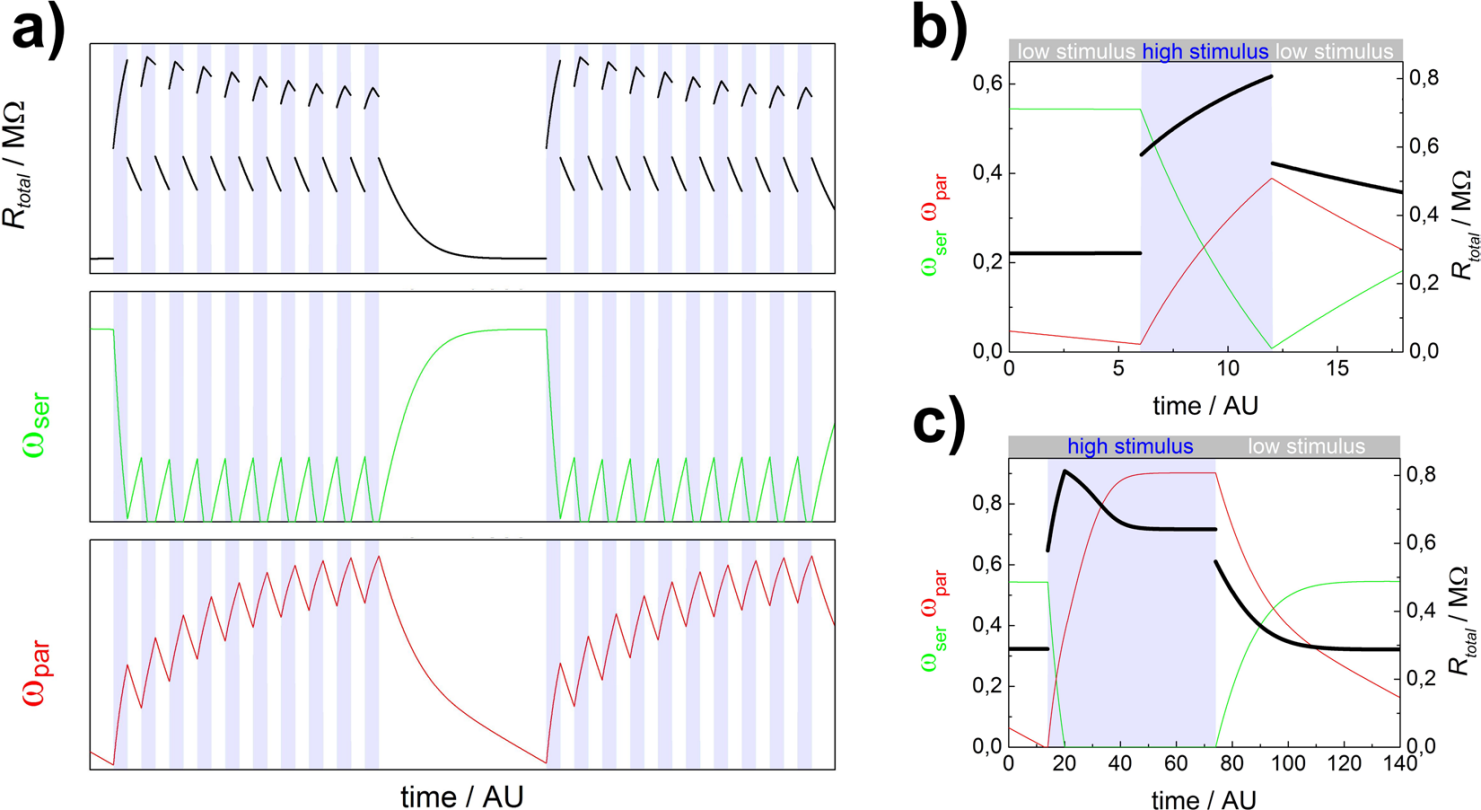
Simulation of adaptation to an external stimulus: (**a**) The response of the memsensor (black line) decreases with each subsequent stimulus pulse (blue background). After a sufficient time at low stimulus, the memsensor recovers. In depth look at the impact of serial (green line) and parallel (red line) memristive element on the memsensor adaptation to external stimulus pulses (blue background). The adaptation is shown at the example of a short pulse (**b**) and a single long pulse (**c**).

Based on the three component model the behaviour of a memsensor with regard to voltage ramps and stimulus pulses was investigated. All parameters for this simulation are given in the supporting material. Comparable results were obtained using the switching model with and without a threshold. For the investigation of the response of a memsensor to stimulus pulses, a constant unipolar voltage is applied to the two terminals. The successful adaptation to a pulsed external stimulus is demonstrated in Fig. 5. The response of the memsensor reaches a maximum value in the first stimulus pulse and decreases asymptotically with each subsequent pulse. After a prolonged low stimulus pulse, the state of the memsensor is restored and its response to the next set of stimulus pulses is identical to the previous set. The step by step calculation in the memsensor model allows plotting the internal states of the memristive devices during the pulses (Fig. 5). At a low stimulus, the parallel memristive device is reset to its high resistive state due to its back driving force. The serial memristive element is at ON state, determined by the equilibrium between the voltage that drops over this element and its internal back driving force. At the high stimulus pulse, the resistance of the sensory element rises and there is a higher voltage drop over the parallel sub-circuit. Accordingly, the state of the parallel memristive element rises and the state of the serial memristive element drops. In the following pulse the memristive elements switch back. At this point one precondition for adaptation as we found in our simulations becomes clear: both memristive elements need to show different relaxation behaviour. As shown in the supplementary data, we chose different time constants and back driving forces to realize this. A similar behaviour can be observed for a single high stimulus pulse for a longer time (Fig. 5c). First the faster serial memristive element drops to its HRS, then the parallel element saturates at a LRS. The overall memsensor resistance reaches its maximum shortly after the onset of the pulse and then gradually adapts to the signal. Especially this behaviour resembles striking similarities to earlier devices from Lupan *et al*. (c.f. first 100 ppm pulse Fig. 4c) and Chiolerio *et al*. (photocurrent measurements, Fig. 2c,d)^6,11^.

**Figure 6:**
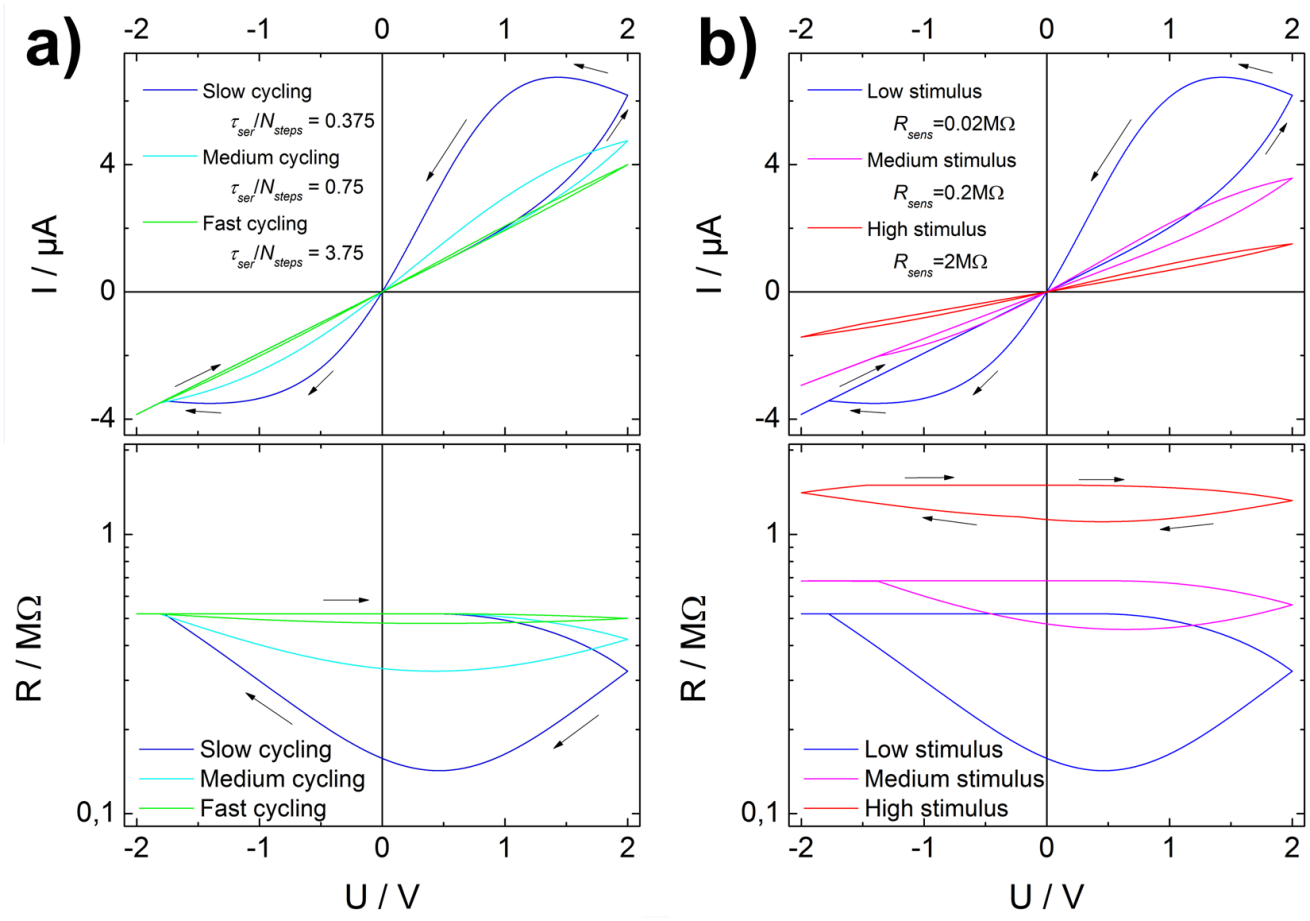
Evaluation of simulated IV hysteresis based on the equivalent circuit. (**a**) Dependency of hysteresis on cycling time, the hysteresis loop narrows for faster cycling times; (**b**) dependency of hysteresis on external stimulus, the hysteresis loop widens for lower external stimulus.

The core feature of a classical memristive device is its pinched current-voltage (IV) hysteresis curve^1,32^. Typically the shape of this hysteresis curve depends on the measurement time. The faster the IV measurement is recorded, the less time the memristive elements have to change their internal state. Accordingly, the hysteresis loop broadens with increasing cycling time^1^. This expected dependency is also observed in the IV characteristic of the memsensor model (Fig. 6a). As the memsensor additionally incorporates a sensitive element, the shape and the extent of the hysteresis loop also depends on the applied external stimulus (Fig. 6b). This dependency is easily explained by considering the equivalent circuit. At zero applied voltage, both memristive elements start in their high resistive state. In case *R*_*sens*_ is low (and *R*_*ser*_ is high), the main voltage drop is over the serial memristive element, which will be switched to its low resistive state until the applied voltages and the back driving voltage are equal. In case *R*_*sens*_ is high (and *R*_*par*_ is high), most voltage will drop over the parallel sub-circuit and accordingly the parallel memristive element will switch to its low resistive state. In addition, the memsensor also shows an offset in its HRS, depending on the applied stimulus. The origin of this offset is the change in resistance of the sensor element by applying a stimulus, which reflects on the overall resistance of the memsensor.

### ZnO rods as a memsensor prototype

A broad variety of materials and sensing signals has been proposed^5–17^. The memristive effect and UV-sensitivity in ZnO nanorods was recently described in detail by Russo *et al*. at the example of Ag/ZnO/FTO structures^8^. Accordingly, we chose Ag/ZnO/Ag structures, in which the ZnO rods are contacted on both sides by silver glue. Typically nanostructures are preferred for their capability of faster sensing. However, we chose bigger structures, namely ZnO microrods for their higher time constants in sensing UV-light, matching better to biological time constants of adaptation.

**Figure 7:**
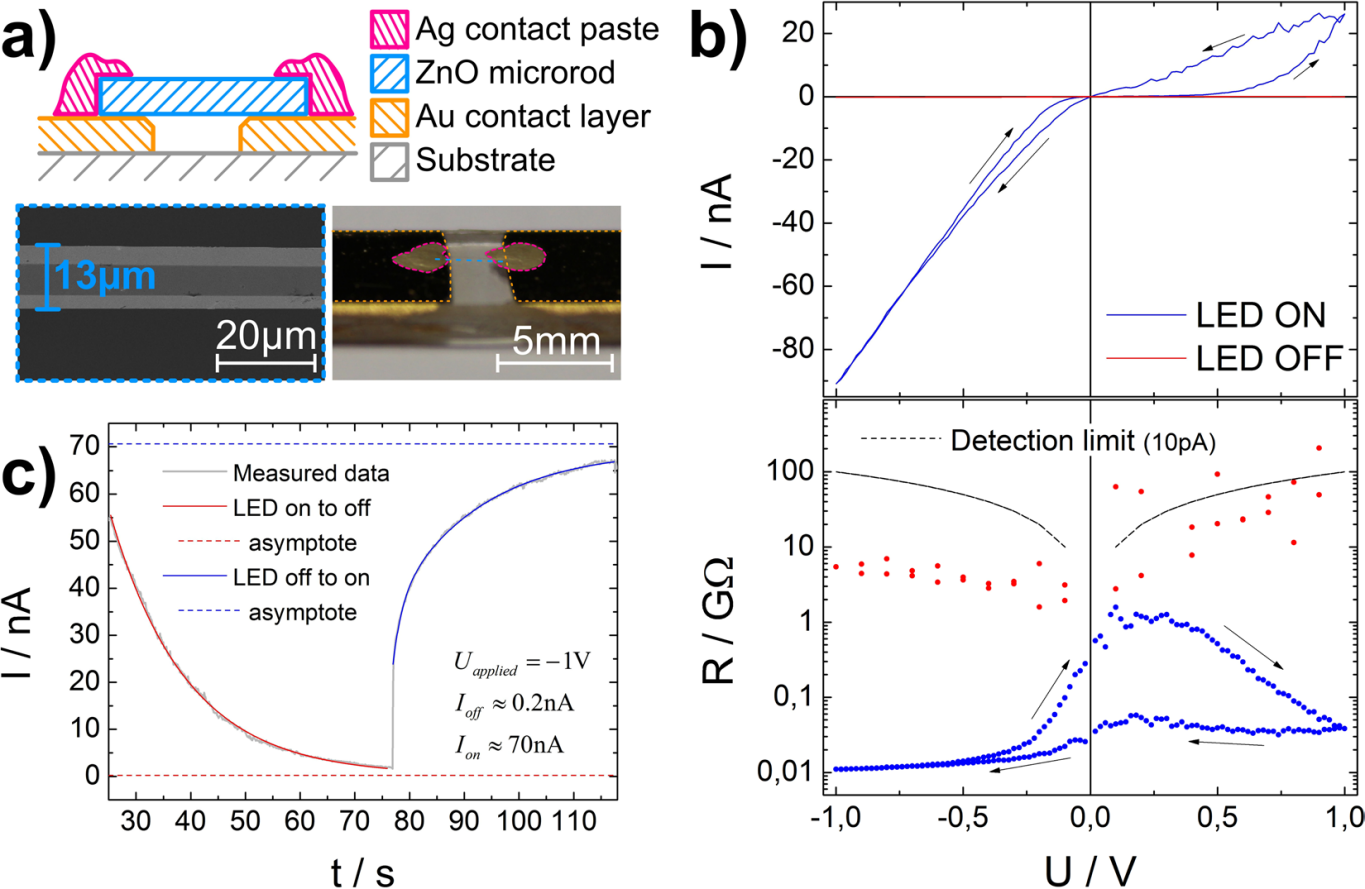
(**a**) Schematic drawing (top) and a photographic image (right) of a ZnO microrod device for comparison. The SEM micrograph is showing the diameter of the ZnO microrod is 13 µm (left); The IV hysteresis curves show strong dependence on the illumination by UV light (**b**) as shown in linear IV plot and logarithmic RV plot. Without UV illumination the ZnO device approached the limit of reliable detection (10 pA). The time constants for the sensing of UV light are determined (**c**) to be in the range of seconds.

In Fig. 7a a typical ZnO microrod device is depicted schematically, the insets show a SEM micrograph as well as a photographic image of a ZnO microrod sample. We prepared ten ZnO microrod samples and recorded IV curves with and without irradiation of UV-light (Fig. 7b). The device yield for working memristive devices is approximately 20% and is mainly limited by handling of the ZnO microrods and contacting them macroscopically with silver glue. For completeness, a full overview over all prepared samples can be found in the supplementary material (Fig. S2).

The memristive switching devices yield a good prototype to showcase stimulus dependent hysteresis as a memsensor feature. To investigate the sensing properties, namely relaxation times for excitation *τ*_*exc*_ and relaxation *τ*_*rel*_ and the sensitivity *S* (the ratio of the device resistance with and without applied stimulus), the time dependence of the ZnO samples overall resistance upon switching of the UV irradiation is recorded (Fig. 7c). The Ag/ZnO/Ag microrod device shows a sensitivity of roughly 350 and the time constants are in the order of seconds to tens of seconds (*τ*_*exc*_ ≈ 11 s, *τ*_*rel*_ ≈ 14 s), which offers good observability and is more close to biological time constants for spike frequency adaptation^25^. The typical µs to ms time constants obtained in classically well performing sensors are somewhat comparable to the time constants of individual neuron spikes^33^.

The IV-characteristics for a memristive switching ZnO microrod show strong dependencies of pinched hysteresis curves on the applied UV light (Fig. 7b). In the UV illuminated state, a single ZnO microrod is a memristive device with an ON/OFF ratio of up to 35. Without UV-stimulus, the rod shows about four orders of magnitude higher resistance, which is partially above the limit of reliable detection in the used setup.

In general, the finding of stimulus dependent hysteresis in our model was also experimentally proven by the ZnO microrod devices. However, the details of the shape of pinched hysteresis curve in simulation and in experiment differ, as our model does not include the nonlinearity of a Schottky contact potentially formed at the contact of the microrod with the silver glue. The Schottky barrier to the silver paste seems to be highly relevant to achieve memristive behaviour. Switching by supplying charges to Schottky interface is well known for memristive devices^4,34^.

### Guidelines for memsensor design

Based on the evaluation of state of art sensitive memristive devices and our investigations on ZnO microrods, we propose three design guidelines to facilitate further research on memsensor devices.

#### The choice of material

In any memsensor, memristive switching has to be joined with sensitivity towards a stimulus. A good starting point for possible memsensor materials are memristive device whose electronic structure of the bulk device or its interfaces with the contacts can be changed by an external stimulus. Metal oxide materials, such as CuO, ZnO, Fe_2_O_3_ or TiO_2_, are promising candidates for memsensor devices, as they combine sensitivity (e.g. towards photons) and memristive properties. For an application in neuromorphic networks, e.g. as feed forward network with external sensitivity, a locally applicable signal is beneficial. Thus light, biomolecules or gases would resemble a better signal than delocalized signals like temperature.

#### Matching properties

The memristive device shows a pinched IV hysteresis as a characteristic property. In order to allow for adaptation, this switching has to be analog. Thus filamentary memristive devices with binary switching are inferior to, e.g., devices in which charges are shifted from and to a Schottky barrier. There is no need for ultrafast switching or sensing. In contrast, for applications as bio-inspired feed-forward networks, the time constants could match biological time constants.

#### The configuration

A prerequisite for any memsensor is its accessibility to the external stimulus. An overview on possible device schematics is shown in Fig. 1. In case of horizontal structures like nanowires, the memsensor is open to the surrounding and thus also the sensory signal. However, large scale integration may render challenging with this device setup. For vertical structures, in which the active layer is sandwiched between two electrodes, integration into cross bar arrays is possible and was already demonstrated. However, a structuring of the top electrode is mandatory in order to make the memsensor accessible to external stimuli like light or gas molecules.

## Conclusion

We report on the concept of memsensors, a class of two terminal devices combining properties of memristive devices and sensors. The junction of features of memristive devices (pinched hysteresis, memory) and sensors (stimulus dependent resistance) gives rise to stimulus dependent IV hysteresis and adaptation to the applied stimulus, which are the fingerprints of a memsensor.

We propose a memsensor model based on three components. In the equivalent circuit, two memristive elements are connected in parallel respectively in series to the sensory element. The model is capable of resembling adaptation behaviour to external stimulus and stimulus dependent hysteresis and thus showcases all features of a memsensor device. The experimental investigation on ZnO microrods replicates stimulus dependent hysteresis.

Based on a review on the state of art in memristive devices with sensing capabilities and our investigations, we proposed three design guidelines to facilitate further research on memsensor devices.

## Methods

### Fabrication of ZnO-microrods as memsensor prototype

ZnO structures were grown using the flame transport synthesis^35^. A mixture of 15 g zinc powder, polyvinyl butyral (PVB), and ethanol was created using a ratio for Zn:PVB:Ethanol of 1:1:2 and subsequently heated inside a crucible to 900 °C for 2 hours. The fast growth in c axis direction leads to the formation of rod shaped micro structures. The diameter of the microrods can range from 1 to 100 µm and with lengths between 200-5000 µm. The structure of individual microrods was investigated earlier using X-ray nanobeam diffraction revealing their single crystalline nature^36^. Single ZnO microrods with diameters of about 13 µm and lengths of about 5 mm were selected manually for the sensor devices. Sputter deposition of gold using a shadow mask was employed to create two contacts with a gap of 3 mm on polycarbonate substrates. The microrods were mounted to bridge the gap between the two contacts and contacted using Acheson 1415 silver.

### Electrical analysis

Current-Voltage (IV) characterisation of the ZnO memsensor systems was done using a Keithley 2400 source meter and a custom made UV-illumination chamber. The sample was placed in a metal unibody case in order to guarantee absence of unwanted illumination. Inside the case, a UV LED (369.7 nm peak, c.f. supporting information) was placed directly above the sample. Pulsing of the UV illumination was achieved by PWM control of the LED constant power source (Meanwell LDD500) and an Arduino Uno.

## Supplementary information

Supporting Information

## Data Availability

The data generated and analysed in this study are shown in the present publication as well as the supporting information and access to specific datasets is available from the corresponding author on reasonable request.

## References

[1] Strukov DB, Snider GS, Stewart DR, Williams RS (2008). The missing memristor found. Nature.

[2] Ielmini D (2016). Resistive switching memories based on metal oxides: Mechanisms, reliability and scaling. Semicond. Sci. Technol..

[3] Edwards, B. A. H. *et al*. Reconfigurable Memristive Device Technologies. **103** (2015).

[4] Wang, Z. *et al*. Nanoionics-Enabled Memristive Devices: Strategies and Materials for Neuromorphic Applications. *Adv. Electron. Mater*. **3** (2017).

[5] Wang X, Chen Y, Gu Y, Li H (2010). Spintronic memristor temperature sensor. IEEE Electron Device Lett.

[6] Chiolerio A (2015). Ultraviolet mem-sensors: flexible anisotropic composites featuring giant photocurrent enhancement. Nano Res.

[7] Li H (2017). Light and magnetic field double modulation on the resistive switching behavior in BaTiO3/FeMn/BaTiO3trilayer films. Phys. Lett. Sect. A Gen. At. Solid State Phys.

[8] Russo P, Xiao M, Liang R, Zhou NY (2018). UV-Induced Multilevel Current Amplification Memory Effect in Zinc Oxide Rods Resistive Switching Devices. Adv. Funct. Mater..

[9] Chen S, Lou Z, Chen D, Shen G (2018). An Artificial Flexible Visual Memory System Based on an UV-Motivated Memristor. Adv. Mater..

[10] Nyenke C, Dong L (2016). Fabrication of a W/CuxO/Cu memristor with sub-micron holes for passive sensing of oxygen. Microelectron. Eng..

[11] Lupan O (2017). Localized Synthesis of Iron Oxide Nanowires and Fabrication of High Performance Nanosensors Based on a Single Fe 2 O 3 Nanowire. Small.

[12] Fan Z, Fan X, Li A, Dong L (2013). Nanorobotic *in situ* characterization of nanowire memristors and ‘memsensing’. IEEE Int. Conf. Intell. Robot. Syst..

[13] Carrara S (2012). Memristive-biosensors: A new detection method by using nanofabricated memristors. Sensors Actuators, B Chem.

[14] Mohamad Hadis NS, Manaf AA, Herman SH (2013). Trends of deposition and patterning techniques of TiO2 for memristor based bio-sensing applications. Microsyst. Technol..

[15] Tzouvadaki I (2016). Label-free ultrasensitive memristive aptasensor. Nano Lett..

[16] Puppo F, Di Ventra M, De Micheli G (2014). & Carrara, S. Memristive sensors for pH measure in dry conditions. Surf. Sci.

[17] Kumar P (2017). Cross-Point Resistive Switching Memory and Urea Sensing by Using Annealed GdO _x_ Film in IrO_x_/GdO_x_/W Structure for Biomedical Applications. J. Electrochem. Soc..

[18] Webster M (2012). Evolving concepts of sensory adaptation. F1000 Biol. Rep.

[19] Adibi M, McDonald JS, Clifford CWG, Arabzadeh E (2013). Adaptation Improves Neural Coding Efficiency Despite Increasing Correlations in Variability. J. Neurosci..

[20] Wang X, Chen Y, Xi H, Li H, Dimitrov D (2009). Spintronic memristor through spin-thorque-induced magnetization motion. IEEE Electron Device Lett.

[21] Ungureanu M (2012). A light-controlled resistive switching memory. Adv. Mater..

[22] Zhu B (2016). Skin-Inspired Haptic Memory Arrays with an Electrically Reconfigurable Architecture. Adv. Mater..

[23] Adrian ED, Zotterman Y (1926). The impulses produced by sensory nerve‐endings: Part II. The response of a Single End‐Organ. J. Physiol.

[24] Maass, W. & Bishop, C. M. *Pulsed Neural Networks*. *Science* 275, (The MIT Press, 1999).

[25] Benda J, Herz AVM (2003). A Universal Model for Spike-Frequency Adaptation. Neural Comput..

[26] Byrne G, Richardson M, Brunsdon J, Patel A (2000). An evaluation of the care of patients with minor injuries in emergency settings. Accid. Emerg. Nurs.

[27] Freeman, W. J. The Physiology of Perception. *Sci. Am*. 264, 78–85 (1991). 10.1038/scientificamerican0291-78 2000483

[28] Ashcroft, N. & Mermin, D. *Solid**S**tate**P**hysics*. (Harcourt, Inc., 1976).

[29] Prodromakis T, Peh BP, Papavassiliou C, Toumazou C (2011). A versatile memristor model with nonlinear dopant kinetics. IEEE Trans. Electron Devices.

[30] Zha J (2017). A general memristor model and its applications in programmable analog circuits. Neurocomputing.

[31] Zha J, Huang H, Liu Y (2016). A Novel Window Function for Memristor Model with Application in Programming Analog Circuits. *IEEE Trans*. Circuits Syst. II Express Briefs.

[32] Chua L (2014). If it’s pinched it’s a memristor. Memristors Memristive Syst.

[33] Tripathy SJ, Savitskaya J, Burton SD, Urban NN, Gerkin RC (2014). NeuroElectro: a window to the world’s neuron electrophysiology data. Front. Neuroinform.

[34] Hansen M (2015). A double barrier memristive device. Sci. Rep.

[35] Mishra YK (2013). Fabrication of macroscopically flexible and highly porous 3D semiconductor networks from interpenetrating nanostructures by a simple flame transport approach. Part. Part. Syst. Charact..

[36] Hrkac SB (2013). Local magnetization and strain in single magnetoelectric microrod composites. Appl. Phys. Lett..

[37] Ignatov M, Ziegler M, Hansen M, Petraru A, Kohlstedt H (2015). A memristive spiking neuron with firing rate coding. Front. Neurosci.

