# Supplementary material for: Concept and modelling of memsensors as two terminal devices with enhanced capabilities in neuromorphic engineering
Source: Sci Rep. 2019 Mar 13;9:4361. doi: 10.1038/s41598-019-39008-5 (PMC6416308; doi:10.1038/s41598-019-39008-5)
Supplement: Supplementary file 1 — Supporting Information [file 41598_2019_39008_MOESM1_ESM.docx]

**Supporting Information**

Concept and modelling of memsensors as two terminal devices with enhanced capabilities in neuromorphic engineering

*Alexander Vahl^1^, Jürgen Carstensen^2^, Sören Kaps^2^, Oleg Lupan^2+^, Thomas Strunskus^1^, Rainer Adelung^2^, Franz Faupel^1^**

^1^ Institute for Materials Science – Chair for Multicomponent Materials, Faculty of Engineering, Christian-Albrechts-University of Kiel, Kaiserstraße 2, D-24143 Kiel, Germany

^2^ Institute for Materials Science – Functional Nanomaterials, Faculty of Engineering, Christian-Albrechts-University of Kiel, Kaiserstraße 2, D-24143 Kiel, Germany

^+^ Affiliation on leave from:

Department of Microelectronics and Biomedical Engineering, Center for Nanotechnology and Nanosensors, Technical University of Moldova, 168 Stefan cel Mare Av., MD-2004 Chisinau, Republic of Moldova

*Corresponding Author*

* Prof. Dr. Franz Faupel, ff@tf.uni-kiel.de, Institute for Materials Science – Chair for Multicomponent Materials, Faculty of Engineering, Christian-Albrechts-University of Kiel, Kaiserstraße 2, D-24143 Kiel, Germany

Simulation parameters

The relevant parameters for the three elements of the equivalent circuit are shown in **Table S1**. For the simulation, the simplified model without threshold voltage was applied.

**Table S1.** Overview over the simulation parameters

| ***Parameter*** | ***Symbol*** | ***Value*** | |
| --- | --- | --- | --- |
| Serial memristive element: Low resistivity state | *R_m.LRS,ser_* | 5 | kΩ |
| Serial memristive element: High resistivity state | *R_m.HRS,ser_* | 500 | kΩ |
| Serial memristive element: Back driving force | *U_b,ser_* | 0.4 | V |
| Serial memristive element: Time constant | *τ_ser_* | 1500 |  |
| Parallel memristive element: Low resistivity state | *R_m.LRS,par_* | 20 | kΩ |
| Parallel memristive element: High resistivity state | *R_m.HRS,par_* | 2 | MΩ |
| Parallel memristive element: Back driving force | *U_b,par_* | 0.11 | V |
| Parallel memristive element: Time constant | *τ_par_* | 2000 |  |
| Sensitive element: Low resistivity state | *R_s,LRS_* | 20 | kΩ |
| Sensitive element: High resistivity state | *R_s,HRS_* | 2 | MΩ |
| Stimulus pulses: Applied voltage | *U_in_* | 0.5 | V |
| Stimulus pulses: Low stimulus | *α_stim,low_* | 0.02 |  |
| Stimulus pulses: High stimulus | *α_stim,high_* | 0.2 |  |
| Stimulus pulses: Calculation steps per pulse | *N_pulse_* | 100 |  |
| Hysteresis: Calculation steps per cycle | *N_hysteresis_* | 400 |  |

Characterization of UV-LED

The spectrum of the UV-LED applied for the IV characterization of ZnO microrods is determined by UV-vis measurement.


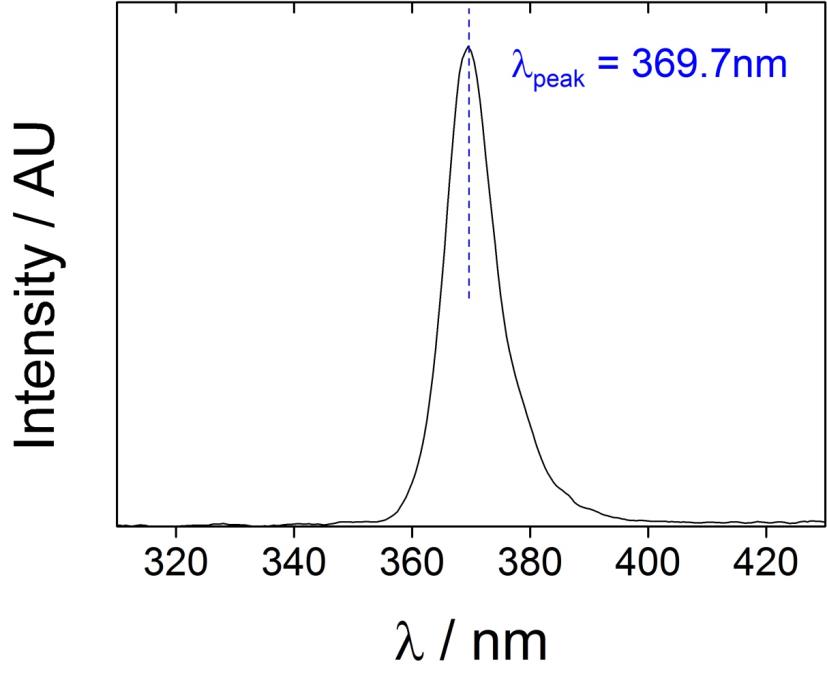


**Figure S1.** Emission spectrum of UV LED as used for testing the stimulus dependency of IV hysteresis in ZnO microrods. The peak wavelength is at 369.7nm.

Systematic overview over series of ZnO rods


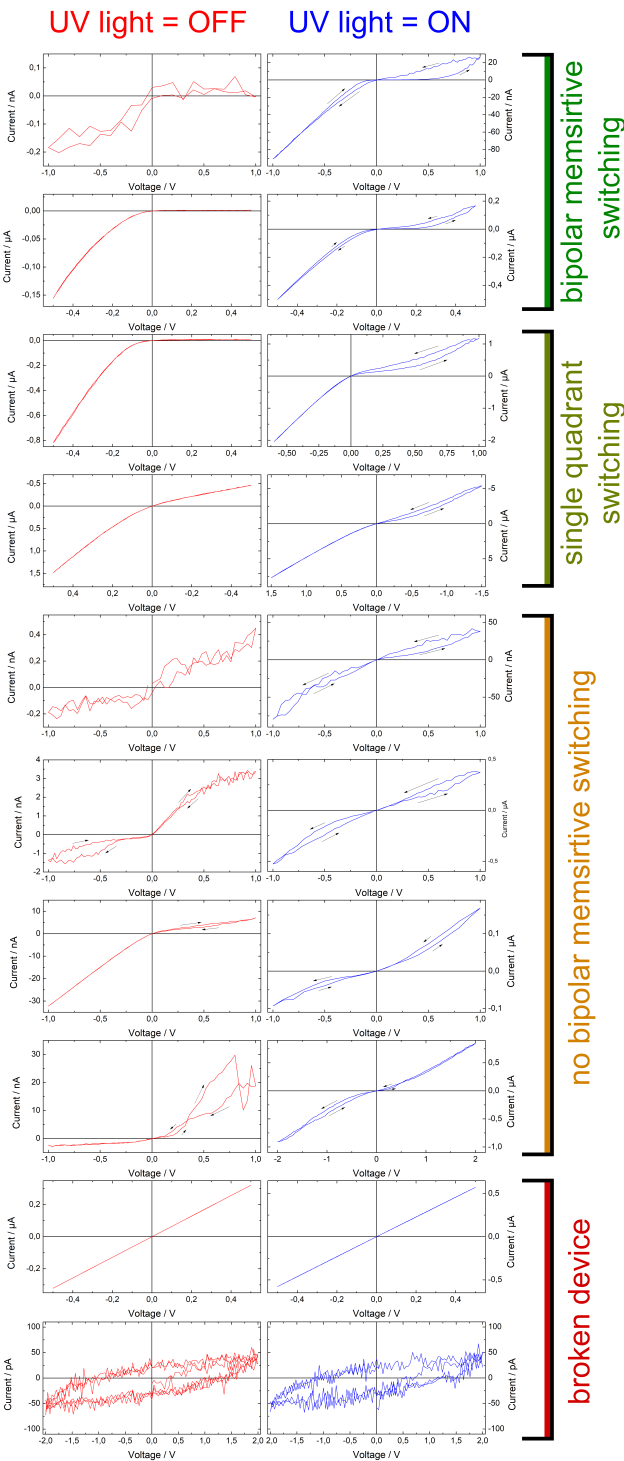


**Figure S2.** Comparison of IV hysteresis for all tested samples, without illumination (red curves) and with UV illumination (blue curve).

Three classes of switching behaviour are observed for the ZnO microrods. Out of ten prepared devices, two showed bipolar memristive behaviour, two showed resistive switching in one quadrant, but no switching for reversed polarity, four samples showed capacitive effects and two samples were broken (either no contact or ohmic contact).
